# Extreme Procoagulant Potency in Human Plasma of Venoms from the African Viperid Genera *Atheris, Cerastes,* and *Proatheris* and the Relative Efficacy of Antivenoms and Synthetic Enzyme-Inhibitors

**DOI:** 10.3390/toxins14120836

**Published:** 2022-12-01

**Authors:** Abhinandan Chowdhury, Matthew R. Lewin, Rebecca Carter, Raul Soria, Matt Aldridge, Bryan G. Fry

**Affiliations:** 1 Venom Evolution Lab, School of Biological Science, University of Queensland, St. Lucia, QLD 4072, Australia; 2 Department of Biochemistry & Microbiology, North South University, Dhaka 1229, Bangladesh; 3 Ophirex, Inc., Corte Madera, CA 94925, USA; 4 California Academy of Sciences, San Francisco, CA 94118, USA; 5 Inosan Biopharma, 28108 Madrid, Spain; 6 MicroPharm Limited, Newcastle Emlyn SA38 9BY, UK

**Keywords:** *Atheris*, *Cerastes*, *Proatheris*, antivenom, marimastat, prinomastat, DMPS

## Abstract

The African viperid snake genera *Atheris, Cerastes*, and *Proatheris* are closely related, similar in size, but occupy extremely divergent ecological niches (arboreal in tropical rainforests, fossorial in deserts, and swamp-dwelling, respectively). Their venoms have not previously been subjected to comparative analyses for their action upon the coagulation of blood, most notably with significant data deficiencies from *Atheris* and *Proatheris.* In contrast, the closely related genus *Echis* is well-documented as capable of producing potent procoagulant effects. In light of this, we set out to compare the coagulotoxic actions of *Atheris ceratophora*, *A. chlorechis*, *A. desaixi*, *A. nitschei*, *A. squamigera*, *C. cerastes*, *C. cerastes gasperettii*, *C. vipera*, and *Proatheris superciliaris* and explore potential pharmacological interventions to reestablish normal blood coagulation. All venoms displayed extremely potent procoagulant effects, over twice as fast as the most potent *Echis* reported to date. Although *Cerastes* is used in the immunising mixture of two different regionally available antivenoms (Inoserp-MENA with *C. cerastes*, *C. cerastes gasperettii*, *C. vipera* and Saudi Arabian polyvalent with *C. cerastes*), none of the other species in this study are included in the immunising mixture of any antivenom. Notably, all the *Cerastes* species were only neutralised by the Inoserp-MENA antivenom. *C. cerastes* venom was not neutralised well by the Saudi Arabian antivenom, with the low levels of recognition for any of the *Cerastes* venoms suggesting a strong regional variation in the venom of this species, as the *C. cerastes* venom tested was of African (Tunisian) origin versus Saudi locality used in that antivenom’s production. The other antivenoms (Micropharm EchiTAbG, ICP EchiTAb-Plus-ICP, Inosan Inoserp Pan-Africa, Premium Serums PANAF Sub-Sahara Africa, South African Vaccine Producers *Echis*, South African Vaccine Producers Polyvalent) all displayed trivial-to-no ability to neutralise the procoagulant toxicity of any of the *Atheris, Cerastes*, or *Proatheris* venoms. Comparative testing of the enzyme inhibitors DMPS, marimastat, and prinomastat, revealed a very potent neutralising capacity of marimastat, with prinomastat showing lower but still significant potency at the same molar concentration, while a 5× molar concentration of DMPS had no apparent effect on procoagulant venom effects normalized by the other inhibitors. These results and methods contribute to the body of knowledge of potential clinical effects and data necessary for evidence-based advancement of clinical management strategies.

## 1. Introduction

Despite snakebite being a well-documented source of significant human morbidity and mortality [1,2], data are lacking for both pathophysiological effects and treatment options for the venoms of many species [3]. Snake venoms exert multi-dimensional attacks upon any part of the body reachable by the bloodstream, and blood coagulation itself is a particular target for many venoms [4]. Coagulotoxic pathophysiological actions not only aid in prey capture, but contribute significantly to the tremendous human snakebite burden.

Within Africa, a diversity of snakes have potent effects upon the blood coagulation cascade. Within the Colubridae family, the sister genera *Dispholidus* (“Boomslang”) and *Thelatornis* are lethally procoagulant through the activation of prothrombin. Treatment options are limited, as only *Dispholidus* is effectively neutralised by Boomslang antivenom [5]. Similarly, within the Elapidae family, spitting cobras in particular have strong anticoagulant effects through the inhibition of the activated clotting factors FXa and thrombin, and neither action is neutralised by South African polyvalent antivenom [6]. However, recent studies have shown that the enzyme inhibitor varespladib is effective against this action [6,7,8]. Within the Lamprophidae family, *Atractaspis* venoms are potently procoagulant through the activation of Factor X, with no antivenom able to effectively neutralise this effect [9].

Significantly, the greatest human impact of coagulotoxic snakebite envenomings in Africa is due to species within the Viperidae family. Of these, the most intensively studied are the venoms of the *Echis* genus, which display extreme procoagulant effects and treatment efficacy suffers from regional variations in antivenom efficacy [10]. Similarly, the coagulotoxic effects of *Bitis* venoms have also been well-studied, with most displaying anticoagulant effects through myriad of mechanisms, except for *B. worthingtoni* which is uniquely procoagulant through the activation of Factor X [11,12,13]. As well, *Causus* venoms have been shown to be anticoagulant through the destruction of fibrinogen, with extreme variation in antivenom efficacy [14]. Within the group of venoms in this study, others have shown that *Cerastes* and *Proatheris* are procoagulant and *Cerastes*, through the activation of Factor X [15]. Though known to cause coagulopathy, hemolysis, thrombocytopenia and renal failure, neither *Cerastes* nor *Proatheris* have been systematically evaluated for relative neutralisation by regionally available antivenoms [16,17,18,19,20,21,22,23,24,25,26]. Few studies have considered the closely related genus *Atheris* [27], though the few data available suggest a strong procoagulant trait with relative in vitro antivenom efficacy remaining unknown prior to the present study [28].

It should be noted that other studies have reported a lack of procoagulant toxicity for *Atheris*, *Cerastes*, and *Proatheris* venoms [29,30]. However, these studies relied upon a laboratory protocol that it did not add the clotting cofactors calcium and phospholipid to the citrated plasmas as part of the methods [29,30,31]. This is an important methodological deficiency as citrate is typically added to plasma to prevent clotting by chelating calcium. Further, platelet poor plasma contains low or no phospholipid as this is contributed in whole blood by the platelets [3]. Not only do many venoms themselves require calcium for activity, but if the venoms activate Factor X even in the absence of calcium, this effect would not be discernible as FX itself requires calcium for its endogenous action of prothrombin activation [5,6,10,32,33,34,35,36,37,38,39,40,41,42,43,44,45]. Thus, while strong procoagulant activity has been reported in a single study of *Atheris* venoms in recalcified plasma [28] and in one physiologically relevant protocol of *Proatheris* [42], the studies which reported a lack of procoagulant toxicity for these two genera (and *Cerastes*) [29,30] must be regarded as potentially erroneous due to likely methodological deficiencies. Similarly a study which reported anticoagulant activity for *Cerastes cerastes* [28] may be due to any number of factors ranging from a regional or ontogenetic variation of the venom to specimen misidentification to methodological issues, with further investigation needed to unravel this issue. These discrepancies [28,29,30] underscore the need for comparative testing in a standardised protocols most closely replicating physiological conditions.

To confirm our hypotheses and ensure methodological consistency with recent standards, we set out to fill some of these knowledge gaps regarding *Atheris, Cerastes,* and *Proatheris* venoms using validated protocols for ascertaining coagulotoxicity. In addition, we also evaluated the relative effectiveness of antivenoms and small-molecule enzyme-inhibitors against the coagulotoxic effects of these venoms [5,6,10,32,33,34,35,36,37,38,39,40,41,42,43,44,45]. We tested the venoms of *Atheris ceratophora*, *A. chlorechis*, *A. desaixi*, *A. nitschei*, *A. squamigera*, *C. cerastes*, *C. cerastes gasperettii*, *C. vipera*, and *Proatheris superciliaris* against regionally available antivenoms (ICP EchiTAb-Plus-ICP, Micropharm EchiTAbG, Inosan Inoserp-MENA, Inosan Inoserp Pan-Africa, Premium Serums PANAF Sub-Sahara Africa, Saudi Arabian polyvalent, South African Vaccine Producers *Echis*, and South African Vaccine Producers Polyvalent) and against several promising enzyme inhibitors 2,3-Dimercapto-1-propanesulfonic acid (DMPS), marimastat, and prinomastat. As all genera have been reported as capable of producing severe clinical effects [16,17,18,19,20,21,22,23,24,25,26], this work will contribute data essential for the evidence-based design of clinical management strategies. This work is also of evolutionary interest as it examines the effect of extremely divergent ecological niche occupation relative to venom effects in related genera such as *Atheris* that are arboreal specialists in tropical rainforests, *Cerastes* species that are semi-fossorial specialists in sandy deserts, and *Proatheris* inhabiting low-lying marshes and flood plains [46,47,48].

## 2. Results

**Figure 1:**
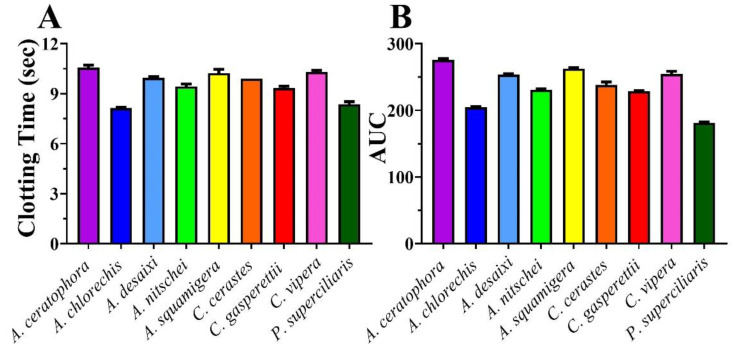
(**A**) 20 µg/mL venom concentration clotting times in human plasma. (**B**) Area Under Curve (AUC) generated from 8-point concentration curves in human plasma; more potent venoms have lower AUCs. Values are mean +/− SD of *n* = 3.

All raw values can be found in the supplementary material in File S1. Supplementary Sheet African viperid genera *Atheris, Cerastes,* and *Proatheris*.xlsx. Plasma clotting conditions were first ascertained by determining the negative control values (spontaneous clotting time subsequent to the addition of calcium), which was 386.5 +/− 13.7 s. Consistent with potent procoagulant toxicity, all venoms tested significantly accelerated the clotting time relative to the negative control (Figure 1). Indeed, the venoms were amongst the fastest viperid venoms to-date tested under the same standardised conditions (only the Indian population of *Daboia russelii* achieved a similar potency (10.4 s) [49]. Notably, at 9.97 +/− 0.6, *A. desaxii* venom is the most potently procoagulant viperid venom tested to-date, a level of potency on par with extremely fast acting Australian elapid venoms in the *Oxyuranus* and *Pseudonaja* genera [44].

**Figure 2:**
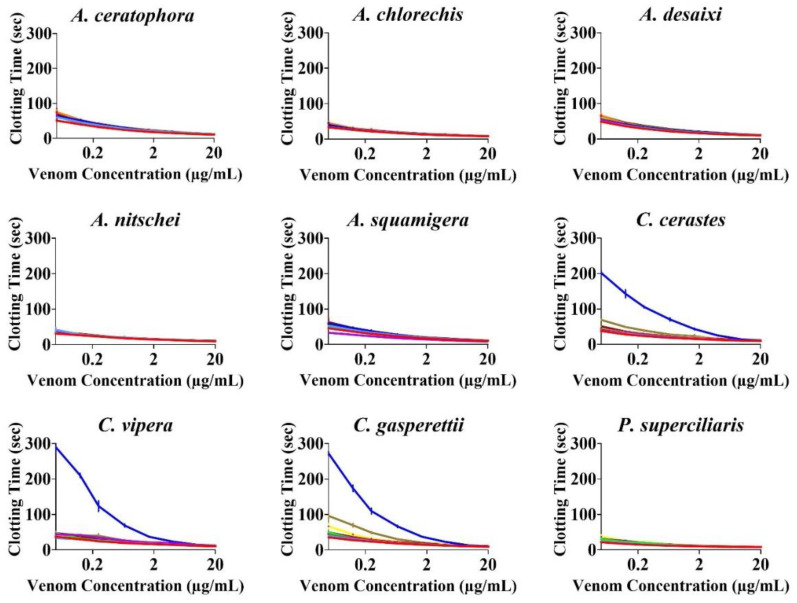
8-point logarithm concentration curves of antivenom against venom. *x*-axis showing concentrations of venom in µg/mL, and *y*-axis showing clotting times in seconds of human plasma. Values are mean ± SD of *n* = 3. Only Inosan’s Inoserp-MENA (dark blue) had any significant effect, and only against *Cerastes* species. The Saudi Polyvalent (brown)_ displayed very low levels recognition of *Cerastes* venoms. Colours are as for Figure 3.

**Figure 3:**
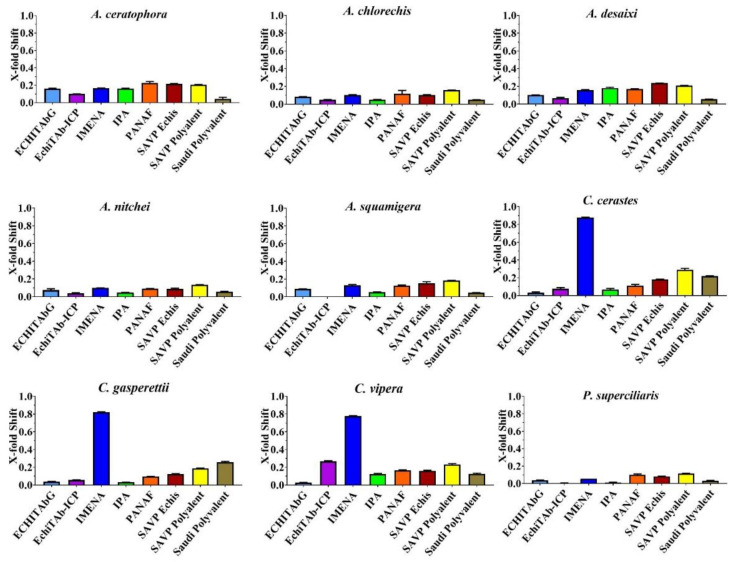
Bar graphs of X-fold magnitude of shift of plasma clotting time due to incubation of antivenoms, calculated by the formula [(AUC of antivenom + venom/AUC of venom) − 1]. A value of 0 indicates no shift (no neutralization by antivenom), while a value above 0 indicates neutralization by antivenom. Values are mean ± SD of *n* = 3. Only Inosan’s Inoserp-MENA (dark blue) had any significant effect, and only against *Cerastes* species.

**Figure 4:**
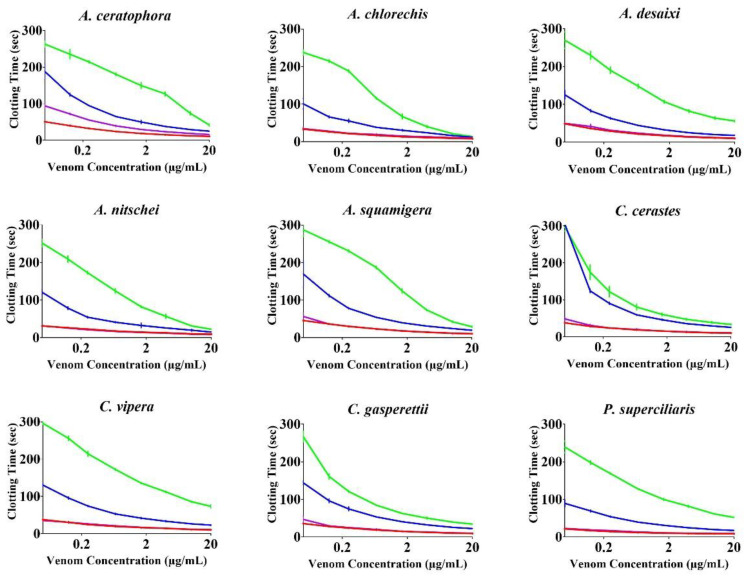
8-point logarithm concentration curves of SMIs against venom. *x*-axis showing concentrations of venom in µg/mL and *y*-axis showing clotting times in seconds of human plasma. Colour legend: Red = Venom, Purple = DMPS (final concentration 1.0 mM %), Indigo = Prinomastat (final concentration 0.2 mM %), Green = Marimastat (final concentration 0.2 mM %). Values are mean ± SD of *n* = 3.

**Figure 5:**
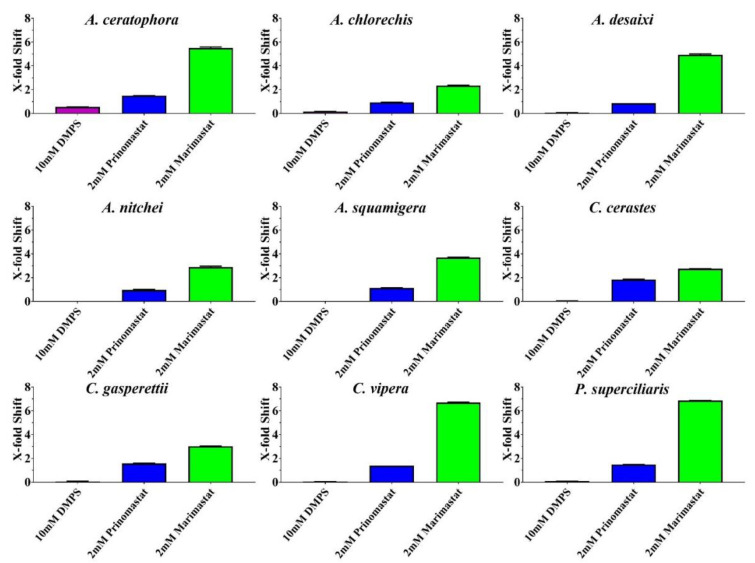
Bar graphs of X-fold magnitude of shift of plasma clotting time due to incubation of SMIs, calculated by the formula [(AUC of SMI + venom/AUC of venom)-1]. A value of 0 indicates no shift (no neutralization by SMIs), while a value above 0 indicates neutralization by SMIs. Values are mean ± SD of *n* = 3. *x*-axis showing SMIs while *y*-axis showing X-fold magnitude of shift.

None of the antivenoms evaluated, with the exception of Inoserp-MENA, which displayed efficacy against all three *Cerastes* venoms, were able to effectively neutralise the other venoms (Figure 2 and Figure 3). The enzyme-inhibitors displayed variation in efficacy. Marimastat consistently outperformed both prinomastat and DMPS (Figure 4 and Figure 5). While the relative efficacy of the three small molecule inhibitors were similar to the results for the procoagulant toxicity of venom from the colubrid snake *Rhabdophis subminiatus*, they are in contrast to results for other viperid snake venoms, where prinomastat showed greater in vitro efficacy than marimastat [32,33,41,42,50]. Conspicuously, even at a 5× concentration, DMPS failed to neutralise any of the venoms, consistent with apparently lesser efficacy seen in vitro against the procoagulant toxicity of other viperid venoms [32,33,41,42,50,51].

As marimastat is a specific inhibitor of metalloproteases, these results suggest that the procoagulant toxicity is due to the presence of snake venom metalloproteases (SVMP) in the venoms. This provides insights into the evolutionary history of their venoms. Specifically, these species are closely related to *Echis,* which use metalloprotease toxins in venom to trigger procoagulant effects in prey, as do the basal *Bitis* species *B. worthingtoni* [52]. This indicates that SVMP-driven procoagulant toxicity is an early arising trait within viperid snakes and that anticoagulant species within this clade (such as [non-*B. worthingtoni*] *Bitis, Eristicophis, Montivipera,* and *Pseudocerastes* other than *P. urarachnoides*) represent derived states. However, future work examining relative effect in activating Factor X versus prothrombin is required to ascertain whether both are basal activities, or if one of these activation strategies is a derived example. A testable hypothesis for such future work is that Factor X activation has been shown to be a widely distributed trait, characterised for *B. worthingtoni, Cerastes*, *Echis*, as a basal trait within the palearctic viper clade *Daboia, Macrovipera,* and *Vipera*, and *Pseudocerastes urarachnoides* [40,52]. In contrast, amongst true vipers prothrombin activation is a much rarer, known only from *Echis* species and *P. urarachnoides*. Thus, it is hypothesised that Factor X activation was present in the last common ancestor of extant true vipers, and that both the prothrombin activation procoagulant trait and the anticoagulant traits are derived activities.

It is important to interpret this work with the appropriate caveats. First is that this work only examined coagulopathy, the antivenom/inhibitor patterns may vary for other pathophysiological effects (such as myotoxicity and neurotoxicty). Secondly, as the work was undertaken under the idealised conditions of preincubating the venoms with the antivenom or inhibitor, future In Vivo testing is required as the next preclinical step before clinical trials can be undertaken to confirm the efficacy of Inoserp-MENA against *Cerastes* venoms or the utility of marimastat against *Atheris, Cerastes,* and *Proatheris* venoms. Reciprocally, the failure of an antivenom or inhibitor to neutralise the venom under idealised circumstances used in this study, strongly suggests that in vivo efficacy would be unlikely in the more dynamic physiological system of a living organism. Thus, except for Inoserp-MENA against *Cerastes,* it is hypothesized that the other antivenoms, and DMPS from within the inhibitors in this study would be unable to neutralise the procoagulant component of the venom induced pathology In Vivo (regardless of any efficacy against other pathophysiological actions such as myotoxicity). Additional studies are thus needed to continue to advance the development of effective treatments to these dangerous venoms.

## 3. Materials and Methods

### 3.1. Stock Preparation

#### 3.1.1. Venoms

All venom work was conducted under the University of Queensland Animal Ethics Approval 2021/AE000075 and UQ Biosafety Committee Approval # IBC/134B/SBS/2015 (Brisbane, Australia for both). Pooled lyophilized venoms purchased from the licensed bioproduct company Latoxan (Valence, France) were: *Atheris ceratophora* (Tanzania), *A. chlorechis* (Ghana), *A. desaixi* (Kenya), *A. nitschei* (Burundi), *A. squamigera* (Sub-Saharan Africa), *Cerastes cerastes* (Tunisia), *C. gasperettii* (Saudi Arabia), *C. vipera* (Egypt), and *Proatheris superciliaris* (Mozambique). Venom stocks were prepared by reconstituting dry venom with 50% glycerol and deionized water to produce 1 mg/mL concentrated stock; stored at −20 °C until further use. Thermo Fisher Scientific™ NanoDrop 2000 UV–Vis Spectrophotometer (Thermofisher, Sydney, Australia) was used to measure the concentration in triplicate at 280 nm wavelength.

#### 3.1.2. Plasma

Frozen human platelet-poor plasma (3.2% citrated) was supplied by the Australian Red Cross (44 Musk Street, Kelvin Grove, QLD 4059, Australia) under research approval #16-04QLD-10. Human plasma work was performed under University of Queensland Biosafety Approval #IBC/134B/SBS/2015 and Human Ethics Approval #2016000256. The plasma was thawed and aliquoted at 1.2 mL quantities, followed by flash-freezing in liquid nitrogen. The aliquots were stored at −80 °C until required for testing. During experiments, these aliquots were defrosted at 37 °C in a Thermo Haake ARCTIC water bath.

#### 3.1.3. Antivenom and Enzyme Inhibitors

Eight regional antivenoms for treating the above viper envenomings were selected (Table 1). Each vial was in 10 mL volume. All the venoms (lyophilized venoms were reconstituted using OK buffer) were centrifuged at 14,000 RCF on Allegra™ X-22R Centrifuge (Beckman Coulter, Brea, CA, USA) at 4 °C for 10 min; the supernatant collected and stored at 4 °C. The antivenoms’ working concentration was 5% (*v*/*v*) using the OK buffer. Code names were given for easier reference.

Small molecule metalloprotease inhibitors tested in this study were, 2,3-Dimercaptopropanesulfonic acid sodium salt monohydrate (DMPS) (catalogue # D8016, Sigma Aldrich) (St. Louis, MO, USA), marimastat ((2S,3R)-N4-[(1S)-2,2-Dimethyl-1-[(methylamino)carbonyl] propyl]-N1,2-dihydroxy-3-(2 methylpropyl)butanediamide) (catalogue # M2699, Sigma Aldrich) (St. Louis, MO, USA), and prinomastat hydrochloride ((S)-2,2-Dimethyl-4-((p-(4-pyridyloxy) phenyl) sulfonyl) -3- thiomorpholinecarbohydroxamic acid hydrocholride (catalogue# PZ0198, Sigma Aldrich) (St. Louis, MO, USA). The powder of these inhibitors were first dissolved in 10% dimethyl sulfoxide (DMSO) and further diluted using deionized water to form 10 mM (prinomastat and marimastat) and 20 mM (DMPS) stock solutions, respectively, and stored at −80 °C. During tests, the working stock of the inhibitors was prepared by further diluting the stock to 2 mM for prinomastat and marimastat and 10 mM for DMPS.

### 3.2. Experimental Conditions

#### 3.2.1. Coagulotoxicity Effects on Plasma

Venom effect on coagulation of plasma was tested by utilising STA-R Max^®^ (Stago, Asnières sur Seine, France) coagulation analyser. From 1 mg/mL venom stock, working stock of 100 μg/mL was prepared by diluting the main stock with OK Buffer (Stago catalogue #00360). Concentration curves (8-points) with serial dilutions of: 1, 1/2, 1/5, 1/12.5, 1/30, 1/80, 1/160, and 1/400 were run by loading the working stock onto the analyser. Inside the analyser, 100 µg/mL working stock was serially diluted to form final reaction concentrations of: 20, 10, 4, 1.6, 0.67, 0.25, 0.125, and 0.05 µg/mL, respectively. Venom stock was added in a cuvette (according to dilution factor) followed by the addition of 50 µL of 0.025 M calcium chloride (Stago catalogue # 00367), 25 µL of OK buffer, and 50 µL of phospholipid (Stago catalogue #00597). The mixture was then incubated for 2 min at 37 °C, and 75 μL of plasma was added immediately right after incubation, and the clotting time was recorded. The whole process is carried out automatically by programmed robotic operation. For positive control, coagulation activator kaolin (Stago C·K Prest standard kit, Stago catalogue #00597) was used, and for the negative control, 50% glycerol/deionized water was replaced with venom.

#### 3.2.2. Antivenom and Enzyme-Inhibitor Efficacy

To investigate the efficacy of antivenoms and enzyme inhibitors in neutralizing the toxic effects of venom upon plasma clotting, the above-mentioned 8-point concentration curves were repeated. In this case, the 25 µL of OK buffer (added to the cuvette before incubation) was replaced with 25 µL of antivenom (0.5% final concentration) or inhibitors (0.2 mM final concentration for prinomastat and marimastat and 1.0 mM final concentration for DMPS). All assays were run in triplicates. Both the experimental conditions were based on validated protocols carried out previously [32,33,41,42,50,51]. 

#### 3.2.3. Statistical Analyses

All data plotting and statistical analyses were done by using GraphPad PRISM 8.1.1 (GraphPad Prism Inc., La Jolla, CA, USA). The area under the curve (AUC) for both venom and antivenom/inhibitors was analysed using the software. X-fold shift was generated using the formulae [(AUC of venom incubated with inhibitors/AUC of venom)—1] utilising Excel. This X-fold shift was scrutinized in a manner where a value of “0” indicated no neutralization (no shift in clotting curve). In contrast, values >0 indicated venom neutralization (change in clotting time curve). Thus, evaluating the activity of the antivenom/inhibitors against venom coagulotoxicity on plasma.

## Figures and Tables

**Table 1:** Antivenom details.

Product Name	Company	Immunising Species	Codes Used in This Study
**EchiTAbG**	MicroPharm Ltd., Newcastle Emlyn, UK	*Echis ocellatus*	ECHITAbG
**EchiTAb-Plus-ICP**	Instituto Clodomiro picado Univerdad de Costa Rica	*Bitis arietans, E. ocellatus, Naja nigricollis*	EchiTAb-ICP
**INOSERP MENA** **(Middle East and North Africa)**	INOSAN Biopharma, Madrid, Spain	*B. arietans, C. cerastes, C. gasperettii, C. vipera, Daboia deserti, D. mauritanica, D. palaestinae, E. carinatus sochureki, E. coloratus, E.s khosatzkii, E. leucogaster, E. megalocephalus, E. omanensis, E. pyramidum, Macrovipera lebetina obtusa, M. l. transmediterranea, M. l. turanica, Montivipera bornmuelleri, Montivipera raddei kurdistanica, Pseuocerastes fieldi, P. persicus, Vipera latastei, Naja haje, N. nubiae, N. pallida, Walterinnesia aegyptia*	IMENA
**INOSERP PAN-AFRICA**	INOSAN Biopharma, Madrid, Spain	*B. arietans, B. gabonica, B. rhinoceros, Dendroaspis angusticeps, Dendroaspis jamesoni, D. polylepis, D. viridis, E. leucogaster, E. ocellatus, E. pyramidum, Naja haje, N. katiensis, N. melanoleuca, N. nigricollis, N. nivea, N. pallida*	IPA
**PANAF** **PREMIUM (Sub-Saharan Africa)**	Premium Serums, Maharashtra, India	*B arietans, B. gabonica, B. nasicornis, B. rhinoceros, Dendroaspis angusticeps, D. jamesoni, D. polylepis, D. viridis, Echis carinatus, E. leucogaster, E. ocellatus, Naja nigricollis, N. haje, N. melanoleuca*	PANAF
**SAVP *Echis***	South African Vaccine Producers	*E. carinatus, E. ocellatus, E. coloratus, Cerastes spp.*	SAVP Echis
**SAVP** **Polyvalent**	South African Vaccine Producers	*B. arietans, B. gabonica, D. angusticeps, D. jamesoni, D. polylepis, Hemachatus haemachatus, N. annulifera, N. melanoleuca, N.mossambica, N. nivea*	SAVP Polyvalent
**Polyvalent Snake** **Antivenom**	National Antivenom and Vaccine Production Centre, Saudi Arabia	*B. arietans, C. cerastes, E. carinatus, E. coloratus, N. Haje, W. Aegyptia*	Saudi Polyvalent

## Data Availability

Data can be found in the supplementary material provided with the paper.

## Supplementary Materials

The following supporting information can be downloaded at: https://www.mdpi.com/article/10.3390/toxins14120836/s1, File S1. Supplementary Sheet African viperid genera *Atheris, Cerastes,* and *Proatheris*.xlsx.
